# Study on the Sequential Combination of Bioethanol and Biogas Production from Corn Straw

**DOI:** 10.3390/molecules24244558

**Published:** 2019-12-12

**Authors:** Katarzyna Kotarska, Wojciech Dziemianowicz, Anna Świerczyńska

**Affiliations:** Institute of Agricultural and Food Biotechnology, Rakowiecka 36, 02-532 Warsaw, Poland; katarzyna.kotarska@ibprs.pl (K.K.); anna.swierczynska@ibprs.pl (A.Ś.)

**Keywords:** lignocellulosic biomass, biofuels, enzymatic hydrolysis, simultaneous fermentation

## Abstract

The objective of this study was to obtain two types of fuels, i.e., bioethanol and biogas, in a sequential combination of biochemical processes from lignocellulosic biomass (corn straw). Waste from the agricultural sector containing lignocellulose structures was used to obtain bioethanol, while the post-fermentation (cellulose stillage) residue obtained from ethanol fermentation was a raw material for the production of high-power biogas in the methane fermentation process. The studies on obtaining ethanol from lignocellulosic substrate were based on the simultaneous saccharification and fermentation (SSF) method, which is a simultaneous hydrolysis of enzymatic cellulose and fermentation of the obtained sugars. *Saccharomyces cerevisiae* (D-2) in the form of yeast cream was used for bioethanol production. The yeast strain D-2 originated from the collection of the Institute of Agricultural and Food Biotechnology. Volatile compounds identified in the distillates were measured using gas chromatography with flame ionization detector (GC-FID). CH_4_ and CO_2_ contained in the biogas were analyzed using a gas chromatograph in isothermal conditions, equipped with thermal conductivity detector (katharometer) with incandescent fiber. Our results show that simultaneous saccharification and fermentation enables production of bioethanol from agricultural residues with management of cellulose stillage in the methane fermentation process.

## 1. Introduction

Starch raw materials (wheat, rye, maize, potatoes) or raw materials containing monosaccharides or disaccharides, i.e., fruit, vegetables, sugar beets, molasses, are used for biofuel production. These raw materials are the basic food for humans and animals, therefore their use for fuel purposes becomes competitive in relation to food production and is the reason for the increase of food prices. An alternative solution is the use of plant raw materials, which are by-products of the agricultural industry and contain lignocellulosic structures—corn straw, rye, barley, and wheat. They are available in larger quantities in comparison with grains and are able to be obtained more cheaply (waste from plant production) [1,2].

The concept of second-generation biofuel development is based on the idea that the raw material used for their production should be a biomass and any organic waste substance [3].

Lignocellulosic biomass is a raw material from the so-called renewable energy source group. The chemical structure and high energy value enable its use in a wide range of conversion processes. It can be used for direct burning, in the production of biogas, or be processed into liquid engine fuels (bioethanol, BTL—Biomass to Liquid) [4,5].

The lignocellulosic complex is of a complicated structure, which is built mainly out of three connected compound groups [2,6,7,8,9,10,11]:

1. Cellulose (30%–60% of total feedstock dry matter)—the major component of plant biomass, a linear polymer of glucose, harder hydrolytic decomposition is connected to its crystalline and amorphous areas;
2. Hemicellulose (20%–40% of total feedstock dry matter)—is a short, highly branched polymer characterized by complex carbohydrate structures (xylose, arabinose mannose, glucose, and galactose) that often create branched chains, it is more prone to hydrolysis than cellulose;
3. Lignin (15%–25% of total feedstock dry matter)—is an aromatic polymer synthesized from phenylpropanoid precursors, ensures plants with impermeability and immunity against attack from micro-organisms and protection from chemical degradation.

The amount of specific compounds of lignocellulose in cell walls varies due to the plant type, kind, and origin.

According to the literature, obtaining of biofuels from lignocellulosic biomass is carried out by implementing four fundamental stages: pre-treatment, enzymatic hydrolysis, fermentation, and separation of fermentation products [8,12,13].

Obtaining high efficiency in the conversion process of polysaccharides to monosaccharides requires the use of additional treatments during the initial processing of the raw material. The main goal is to remove lignin and perform a hydrolysis of the hemicellulose polymers which, on the one hand, limits an open access of cellulolytic enzymes to cellulose microfibrils and, on the other hand, acts as an absorbent, binding enzyme molecules on its surface, causing their irreversible inactivation [14,15,16].

Literature data indicate the existence of many factors limiting the hydrolysis of lignocellulosic waste. One of them is the crystallinity of cellulose, and others are the degree of polymerization and the availability of surface area and lignin content [4,17]. This is due to the molecular structure (the presence of â-glycosidic bonds) of cellulose fibers. The literature indicates a significant influence of the crystalline structure of cellulose on biomass recalcitrance, manifested by difficulties in the degradation of these polymers [12,18].

The hydrolysis process of the lignocellulosic compound is the most difficult stage in biomass degradation, because the cellulose microfibers are stabilized by external and internal hydrogen bonds and are surrounded by hemicellulose polysaccharides (mannans and xylanes)—connected with hydrogen and covalent bonds [14,19]. In recent years, there has been an interest in studies regarding chemical methods of degradation of individual lignocellulosic structures, resulting also in an interest to maximize the efficiency of bioprocesses [7,20].

Pretreatment methods are either physical, chemical, or biological. However, some methods include both effects. For the purposes of classification, steam and water are excluded from being considered chemical agents for pretreatment since extraneous chemicals are not added to the biomass. Physical pretreatment methods include comminution (mechanical reduction in biomass particulate size), steam explosion, and hydrothermolysis. Comminution, including dry, wet, and vibratory ball milling [21], and compression milling are sometimes needed to make material handling easier through subsequent processing steps. The most commonly used acid and base are H_2_SO_4_ and NaOH, respectively.

Anaerobic digestion is a complex biochemical degradation process, in which organic substrates are decomposed by bacteria forming gaseous by-products, mainly including methane (CH_4_) and carbon dioxide (CO_2_). Under optimal conditions, the biogas contains: methane 52%–85%, carbon dioxide 14%–48%, hydrogen sulfide 0.08%–5.5%, hydrogen 0%–5.5%, carbon monoxide 0%–2.1%, nitrogen 0.6%–7.5%, oxygen 0%–1%. The composition of the fermentation gas depends mainly on the type of substances that are decomposed in the fermentation chamber [22]. Methane fermentation is divided into several stages, each of which takes place with the participation of many groups of interdependent microorganisms. Four phases can be distinguished: hydrolysis, acidification, acetate, and methanogenic phase.

The research was aimed at obtaining bioethanol and utilizing the post-fermentation residue for obtaining the biofuel, which is biogas. Thus, the waste product from the first process was the raw material for the second production.

The concept of waste biomass utilization in a sequential combination of two biochemical processes—ethanol fermentation and methane fermentation—will be important in specific benefits relating to environmental protection: the reduction of organic pollution, utilization of excess waste from the agricultural sector, and reduction of dust and gas emissions from conventional energy sources combustion.

## 2. Results and Discussion

### 2.1. Pretreatment and Chemical Composition of Corn Straw

**Figure 1:**
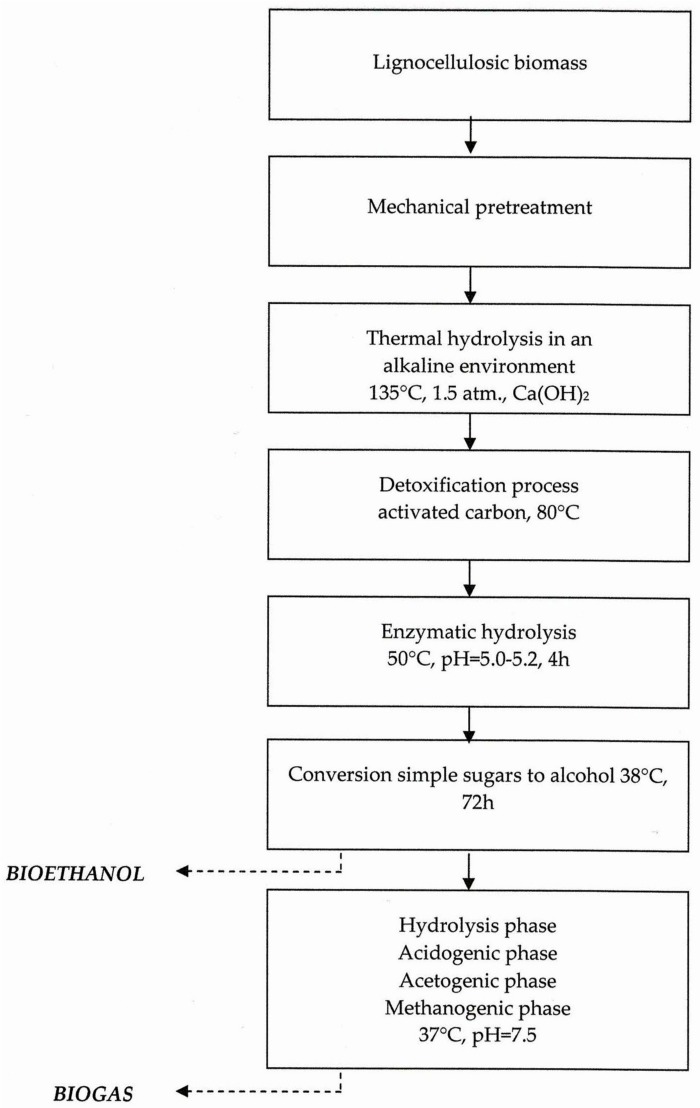
Flowchart of bioconversion of lignocellulosic biomass to second-generation biofuels.

The processes to obtain biofuels of lignocellulosic biomass (corn straw) were performed according to the procedure presented in Figure 1 and described quantitatively in Section 3.2:

- initial processing—mechanical fragmentation of biomass, thermal hydrolysis, detoxification;
- enzymatic hydrolysis;
- alcoholic fermentation;
- methane fermentation.

By performing alkaline sample processing, the material was exposed to calcium hydroxide and then to pressure and thermal processing. According to the literature and preliminary research, it had been found that two-stage pretreatment, i.e., thermal hydrolysis in an alkaline environment and enzymatic hydrolysis, results in a high level of polysaccharide conversion. Enzymatic hydrolysis is reported as the most promising technique for converting lignocellulosic compounds into fermentable sugars such as glucose, which can be used as a cheap carbon source for ethanol production. The main role of alkali in biomass pretreatment is the removal of lignin, concomitantly increasing enzyme effectiveness by eliminating non-productive adsorption sites, and increasing the accessibility of the enzyme to structural carbohydrates [23]. This explains the difference in the rate of bioconversion observed between the alkali combined in sequence with enzyme and without enzyme pretreatment. The conducted preliminary research of lignocellulose decomposition showed that the use of enzymatic degradation of corn straw resulted in an increase in the content of simple sugars in the raw material by 85% in relation to the pretreatment without enzymatic hydrolysis.

It was determined that during thermal hydrolysis, toxic compounds are produced—which inhibit microorganisms that cause alcoholic and methane fermentation. Those compounds can be divided into four groups: sugar decomposition products, lignin decomposition products, compounds created from lignocellulose compound decomposition, and heavy metal ions. Inhibitor presence decreases both the efficiency and fermentation speed, which results in processing time prolongation. To remove the inhibiting compounds, additional detoxification process was conducted before applying the enzymatic preparation.

Simultaneous activity of two enzymatic preparations was used for polysaccharide decomposition: Celluclast 1.5 L (cellulase) and Viscozyme Wheat HT (arabanase, β-glucanase, hemicellulase, and xylanase). According to the literature, the most effective enzyme compound is a set of cellulase, xylanase, and cellobiase, which attests to their synergetic working. Using additional enzymes with cellulose helps increase the effectiveness of polysaccharide hydrolysis of lignocellulose biomass [24,25].

The efficiency of initial processing and enzymatic hydrolysis was defined based on, among the others, fiber content: neutral detergent (NDF), acid detergent (ADF), and acid detergent lignin (ADL) in corn straw [26].

The content of crude fiber in dry corn straw was 39.21%. The use of initial processing and enzymatic hydrolysis (before fermentation) reduced the amount of crude fiber to 12.15%. The efficiency of cellulose hydrolysis to simpler compounds is limited mostly by the presence of hemicellulose and lignin—polymers that limit a free access of cellulosic enzymes to cellulose microfibrils. The content of basic fractions (cellulose, hemicellulose, and lignin) in the dry mass of corn straw was respectively: 11.57%, 43.21%, and 2.22%, Table 1.

By implementing initial processing and enzymatic hydrolysis, the amount of lignin, cellulose, and hemicellulose decreased by 43%, 33%, and 78% respectively. The best results were obtained for two-stage processing, containing alkaline and enzymatic hydrolysis. This proves the sound use of an additional stage of chemical hydrolysis of the cellulose biomass, resulting in increased overall efficiency of the alcoholic fermentation process.

### 2.2. Ethanol Fermentation

After the initial processing and detoxification of the lignocellulose raw material, it was subjected to enzymatic processing and alcoholic fermentation.

The studies on obtaining ethanol from lignocellulosic substrate were based on a process called simultaneous saccharification and fermentation (SSF). When both processes are carried out in the same reaction vessel, enzyme inhibition can be prevented, while the sugars released are rapidly converted by the microorganisms. The conditions for SSF have to be a compromise between the optimal conditions for hydrolysis and fermentation. The cellulases work optimally at pH 4–5 and temperatures of 40–50 °C, whereas *S. cerevisiae* (hexose fermentation) ferments optimally at 30 °C and pH 4–5 and the pentose-fermenting organisms work optimally at 30–70 °C and pH 5–7. The conducted preliminary research showed that the use of the simultaneous saccharification and fermentation (SSF) and separate hydrolysis and fermentation (SHF) methods results in a similar alcohol yield. The SSF method was chosen due to the shorter time of the ethanol production process and the elimination of the negative effect of mono- and oligo-saccharides on enzymatic hydrolysis, as compared to the SHF method. The process of simultaneous saccharification and fermentation using *Saccharomyces cerevisiae* (D-2) yeast was performed in 38 °C, 40 °C, and 42 °C temperatures. Alcohol efficiency from lignocellulose biomass in 40 °C temperature was higher by 9% in comparison to the 38 °C temperature, and higher by 27% in comparison to the efficiency recorded in the 42 °C temp. That is why the research presented in this article was associated with the alcoholic fermentation of lignocellulose biomass conducted in 40 °C temperature. There are multiple published studies related to SSF fermentation of lignocellulose biomass, using *Saccharomyces cerevisiae* yeast in 30–40 °C temperatures [27,28,29].

The apparent extract, which at the beginning of fermentation was ca. 5 °Blg, decreased every day to the respective values: 3.8 °Blg (24 h) and 2.9 °Blg (48 h and 72 h), Table 2. During the alcoholic fermentation process, no contamination caused by bacteria was detected, which is confirmed by the pH of mash equaling 4.6 ± 0.1, marked after 24, 48, 72 h. There was no increase in hydrogen ions during the test—which is usually characteristic of acetic fermentation caused by bacteria. After 72 h of the alcoholic fermentation process, the alcohol concentration was 16.98 g/L. The content of directly reducing sugars in cellulose stillage obtained from corn straw fermentation was in the range of 6.12–4.90 mg/L (24–72 h), Table 2.

This low amount of reducing sugars attests to a positive course of the fermentation process and to the utilization of monosaccharides available in the base by the *Saccharomyces cerevisiae* yeast. The content of sugars was reduced by 74% in correlation to the initial sugar level—19.10 mg/L.

Based on the obtained alcohol concentration, biotechnological indicators of the fermentation were calculated—the ethanol yield from cellulose, actual speed of fermentation, fermentation productivity, and yield. The obtained results of biotechnological indicators of alcoholic fermentation are presented in Table 3.

By analyzing the examination results, it can be observed that the ethanol yield was 31.25 L EtOH/100 kg of cellulose. The speed of actual alcoholic fermentation and productivity of alcoholic fermentation in the 72nd hour of the process were, respectively, 3.91 L EtOH/kg of glucose x h and 0.30 L EtOH/L of mash x h, Table 3.

When comparing alcohol efficiency in individual days with other biotechnological indicators, it can be determined that the alcoholic fermentation of lignocellulosic biomass was very intense in the first two days of the process. During the third day, there was a significant decrease in fermentation speed and efficiency, which correlated with the same results for alcohol efficiency obtained in the second and third day.

Based on the analysis of biotechnological parameters and indicators, it was determined that the alcoholic fermentation process was properly conducted.

When the fermentation process ended (72 h), alcohol was distilled from the examination samples to determine its purity. In the conducted GC study, higher alcohols—methanol, aldehydes, acrolein, and furfural—were indicated. It was determined that the amount of aldehydes was at 0.209 g/L EtOH and it was comprised of acetaldehyde, propionaldehyde, and 2-furaldehyde (furfural), Table 4.

In the examined spirits, there was no trace of acrolein, which has cancerogenic properties. Its presence causes a detectable worsening of the spirit’s sensory characteristics.

The amount of higher alcohols produced in the examined samples was low and equaled 2.009 g/L EtOH. When the bioethanol is used as a fuel biocomponent, the value of higher alcohols cannot exceed 2% [*v*/*v*]. Also, a low amount of methanol was detected in the examined raw spirit samples, i.e., 0.001 g/L EtOH.

One of the challenges related to the production of ethanol from floral biomass is the use of all waste products created during the process. Only by doing so can the process be profitable and ecologically balanced. One of the solutions is to utilize the waste from the production of bioethanol from lignocellulose mass to produce biogas. Additional beneficial trade is the possibility to use the post-fermentation created during biogas production as a fertilizer in agriculture [30,31].

### 2.3. Methane Fermentation

After successful alcoholic fermentation of lignocellulose biomass and bioethanol production, the remaining cellulose stillage was subjected to methane fermentation.

The whole stillage from ethanol fermentation was directly transferred to an anaerobic digester and characterized by a dry matter (DM)—92.0 g/L, dry organic matter (DOM)—66.2 g/L DM, ash content—28.04 g/L DM, total nitrogen—0.16%, chemical oxygen demand (COD)—38.45 gO_2_/L, reducing sugars—0.51%, ethanol—not determined and volatile fatty acids (VFA)—850 mg/L.

**Figure 2:**
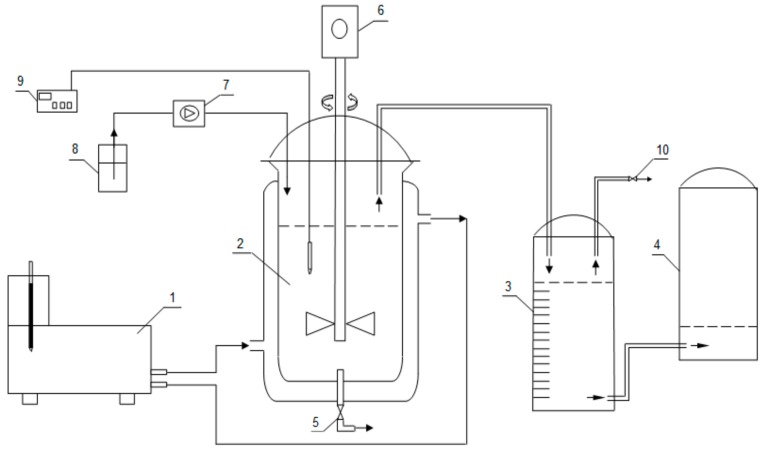
Experimental set-up. **1**—Thermostat; **2**—Fermentation chamber; **3**—Tank for measurement of biogas volume; **4**—Tank compensatory pressure; **5**—Valve for fermentation medium fetch; **6**—Stirrer; **7**—Peristaltic pump; **8**—Tank for NaOH solution; **9**—pH meter; **10**—Valve for biogas fetch.

Biogas production was carried out in an experimental set-up of the anaerobic digestion process, which was presented in Figure 2.

The examination focused on periodical (static) fermentation—conducted in one hermetically sealed container, with no air flow. The setup consisted of a continuous stirred bioreactor 2 L (1.5 L working volume), equipped with temperature regulation, and a water displacement system for biogas collecting. A single-blade type agitator was introduced into the reactor. In order to reduce the cutting force while stirring the contents of the reactor, openings on both sides were cut out in the mixer. The size of the blades was chosen in such a way that even at very low speeds it was possible to stir the fermentation biomass in its entire volume. The mechanical agitator provides more area of contact with the biomass, thus enhancing gas production. The reactor with flat flange and four lateral angled necks on the top was placed on a metal stand. The necks were used for inserting the agitator shaft inside the tank and dispensing the agent, regulating the acidity of the environment.

The reactor pH was maintained at 7.5 ± 0.7 by sodium hydroxide, which was supplied to the fermenter by a peristaltic pump. At the bottom of the reactor there was a valve for sampling the fermentation medium intended for the determination of fermentation parameters.

The tested substrates were placed in the reactor and then flooded with a sufficient amount of inoculum. Anaerobic fermentation processes were conducted under mesophilic conditions at 37 °C. A constant temperature was kept inside the fermentation chamber by a thermostat connected to its water jacket. According to literature, any changes in temperature may cause domination of the acidogenic phase and inhibition of the last methanogenic phase in methane fermentation. A small variation in the temperature of the fermenter affects the biological activity of anaerobic bacteria [32]. Constant temperature is important for preventing negative effects on biogas production. Biogas produced in the fermenter was transferred to a cylindrical store—equalizing reservoirs, filled with a liquid resistant to gas solubility.

The cellulose stillage produced during alcoholic fermentation of corn straw was characterized by a dry matter (DM) content of 92.0 g/L and dry organic matter (DOM) of 66.2 g/L DM. This level of extract content is optimal for the anaerobic digestion process and enables the stirring of the medium during the process course.

The following parameters of methane fermentation that can influence the process stability were marked during a 24 h cycle: pH, dry matter, dry organic matter, mineral substances, and VFA’s (acetic acid, propionic acid, and butyric acid), Table 5. In addition, daily volume growth for the produced biogas was measured. Moreover, the flammability of the biogas was marked in a 48 h cycle to determine its energy value.

It has been determined that the waste created after ethanol production is characterized by good access to biodegradable compounds for the methane bacteria. This results in a rapid acid phase and efficient conduction of subsequent stages of methanogenesis. It has been determined that the periodical methane fermentation process with pH correction of the cellulose stillage can be efficiently conducted for 8 days. After that time, the process slows down and the production of biogas decreases. An initial alcoholic fermentation, which transformed glucose particles into ethanol, influenced the activity time of methane bacteria.

**Figure 3:**
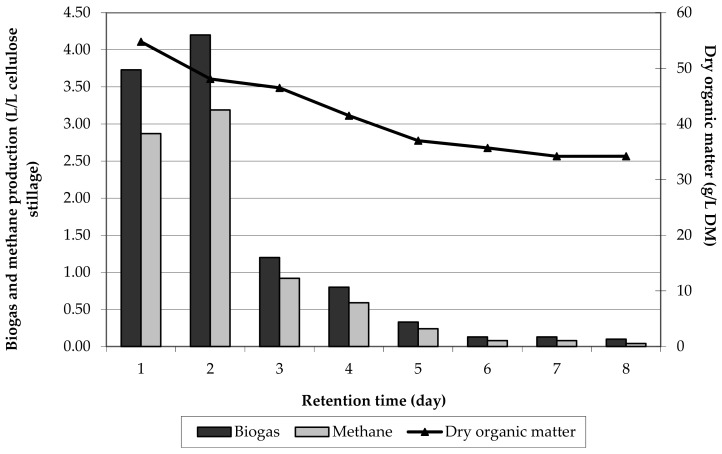
Daily biogas production and dry organic matter content of lignocellulosic biomass, T = 37 °C, pH 7.5.

The highest dry mass reduction and biogas efficiency were observed in the first days of the process. It was the most active period of methane fermentation, during which organic substances were transformed into volatile fatty acids, alcohols, aldehydes, and CO_2_ and H_2_ gas products, Figure 3. The basic action against VFA increase, and subsequently a significant drop in pH occurs, was the addition of NaOH to the fermentation.

The concentration of organic mass decreased during methane fermentation from 66.2 g/L (0 h) to 34.2 g/L, Figure 3. Based on parameters such as dry matter, dry organic matter, and mineral substances, a degree of organic substance decomposition (DOSD) was calculated and equaled 48.34%.

The efficiency of the fermentation process was measured by determining the fermentation module. The most important are the ranges, meaning if the fermentation module is higher than 50% (M_f_ ≥ 50%), then it is assumed that the fermentation degree was correct, and that methane fermentation was running in a stable manner. In this study, the fermentation module was ca. 59% (Table 6), which indicates a correct fermentation degree.

After methane fermentation, 10.63 L of biogas from 1 L of the cellulose stillage was obtained, which comprised ca. 330 L/kg of DOM, Table 6. The biogas production during methane fermentation of corn straw is in the range from 0.20 to 0.60 m^3^/kg of the total volatile solids inserted into the anaerobic fermentation chamber [33,34].

Many factors impact the amount of methane produced: the quality and type of the used substrates, water concentration in the mixture, the temperature of the process, fragmentation degree and substrate fermentation degree, and the used hydraulic activation time. The chromatographic examination showed methane concentration between 68%–77%, while the maximum value occurred along with the highest biogas concentration. The obtained biogas was characterized by high energy value, which was confirmed by the flammability tests. After each day, the produced biogas was burned in a control burner. High methane concentration meant that there were no difficulties in keeping the flame alive.

The cellulose stillage produced during alcoholic fermentation of corn straw was characterized by high COD, i.e., 38.45 gO_2_/L. Conducting methane fermentation allowed to reduce the index by 57% (i.e., 16.43 gO_2_/L).

It was determined that the high level of polysaccharide conversion into simple sugars in corn straw can be obtained by two-stage pretreatment, containing alkaline and enzymatic hydrolysis. Pang [35] showed that when using 6% NaOH (sodium hydroxide) in the pretreatment of corn straw, the biogas yield increased by 48.5%, compared to the variant in which the raw material preparation was not carried out.

It is very important, in particular, to reduce the size of raw material before the pretreatment was carried out. The aim of the mechanical processing is to maximize mass and heat transfer during downstream hydrolysis, reduce crystallinity and shearing, and increase the overall surface area, greatly increasing the total hydrolysis yield. According to the literature, decreasing the size of straw particles to 53–149 mm allowed for increased levels of released glucose and xylose after 24 h enzymatic hydrolysis by 39% and 20%, in comparison to the reference samples [25,36,37]. Sharma et al. [38] showed that the production of biogas using agricultural and sylvan waste (e.g., wheat, rice, grass straw) increased along with the decreased size of the grain from 30 to 0.088 mm.

It was determined that by implementing the detoxification of corn straw, it is possible to obtain higher ethanol concentration by circa 40% and increase biogas production [13]. According to Kumar et al. [39], commercial charcoal resulted in a 17% and 34.7% increase in biogas in batch and semi-continuous fermenters, respectively.

Talebnia et al. [40] studied different microorganisms used in ethanol production. It was determined that the best solution was using *Saccharomyces cerevisiae* yeast. During bioethanol production from lignocellulose materials, it is important for the microorganisms to be resilient to low pH, high temperature, and the presence of inhibitors in the fermentation environment. Resilience to the high temperature of the process is especially significant due to the higher temperature during the conduction of enzymatic hydrolysis. To use both processes simultaneously, i.e., alcoholic fermentation and enzymatic hydrolysis, the fermentation temperature should be as close to the optimal hydrolysis temperature as possible.

Geng et al. [41] conducted research on obtaining ethanol from horticultural waste. The raw material was subjected to pretreatment (using the Organosolv method), enzymatic hydrolysis (using Celluclast 1.5 L and Novozym 188 enzymatic preparations), and alcoholic fermentation using *Saccharomyces cerevisiae* yeast. The research led to producing 11.69 g/L ethanol. Bondesson et al. [42] described the ethanol and biogas production after steam pretreatment of corn stover. The highest concentration of ethanol, 22.6 g/L, was obtained following sulfuric acid pretreatment at 200 °C for 10 min.

In this paper, the efficiency of biogas was ca. 330 L/kg of DOM, while methane efficiency was ca. 250 L/kg of DOM. Kaparaju et al. [43] described the production of bioethanol, biohydrogen, and biogas from wheat straw subjected to initial processing. The biomass, which was initially processed, was subjected to enzymatic hydrolysis and fermentation processes into ethanol and biohydrogen. The wastes from both processes were supposed to be used in methane production with the following efficiency: 324 and 381 L/kg of volatile solids. In the research by Dererie et al. [44], it was determined that the amount of obtained ethyl alcohol and biogas are dependent on the type of pretreatment. The following pretreatments were used: lime pretreatment, dilute acid impregnation, and steam explosion pretreatment and steam explosion pretreatment. It was found that methane yield from oat straw is in the range from 260 L to 300 L/kg of volatile solids.

A strong tendency for lowering the pH of methane fermentation substrate and increasing volume of volatile fatty acids (VFA) were noticed. The obtained results are confirmed by the study of Kacprzak et al. [11], who examined the efficiency of biogas production from energy crops (phalaris). They concluded that biogas production was the highest in the first days but decreased and was not regular after the third day. VFAs are key intermediates in the biomethanation process that are capable of inhibiting methanogensis at high concentration [19]. Pullammanappallil et al. [45] reported the digester failure when the concentration of acetic acid and propionic acid reached above 3000 mg/L. High VFA inhibits growth of acid producing bacteria, thus reducing the rate of acidogensis. Fermentation of sugar is inhibited by total VFA concentrations above 4 g/L [46,47].

Joelsson et al. [48] described the production of ethanol and biogas from acetic acid-impregnated steam-pretreated wheat straw. The authors performed a techno-economic evaluation of a biorefinery concerning ethanol, raw biogas, pellet, and electricity production at various solid contents and residence times in the fermentation steps. The overall energy efficiency for the products (in terms of the energy content in the products divided by the incoming energy added to the process) varied from 68% to 72%. Shafiei et al. [49] showed that the overall energy efficiency of the combined biofuels from spruce wood was 79%. The energy efficiency was calculated by dividing energy output of the products (ethanol, solid residues, and methane) with the energy requirements of the process as well as the raw materials. The total energy input of the base case was 169 MW in the form of the wood, electricity, and steam, and the total output was 134 MW in the form of ethanol, lignin, and methane.

Patel [50] studied the biogas and bioethanol production from the cotton stem waste. He reported that average COD removal efficiency was 26.08%, which is comparatively lower than the commonly obtained COD removal from cattle manure (51%–79%). This result was believed to be due to the high COD exhibited by the inoculums in effect. It dominates the microbial activity thereby resulting in lower COD removal efficiency.

According to Magrel [51], after the methane fermentation process, the COD value for slurry and dairy sludge was similar and amounted to 20–30 gO_2_/L. The average COD reduction effect during the fermentation process was from 40% to 60%.

## 3. Materials and Methods

### 3.1. Raw Material

The research material included corn straw (leaves and stems). Table 7 represents the characteristics of the material used in this research.

### 3.2. Pretreatment of Raw Material

#### 3.2.1. Mechanical Comminution

The first stage was the comminution and grinding of the raw material (corn straw). Special cutting shears were used for crushing to change the structure of the raw material and adjust the size of the crushed raw material to its grinding. In the next stage, the raw material was milled using a cutting mill to a particle diameter in the range of 0.12–0.15 mm. The aim of the mechanical processing was to reduce the size of solid parts of the substrate and partial destruction of the crystal structure of the cellulose. It allows the enzymes to more easily access the polysaccharide compound and increased the substrate’s sensitivity to chemical agents [35].

#### 3.2.2. Chemical Pretreatment

The corn straw was pretreated by alkali pretreatment and thermal hydrolysis. Raw material was processed in alkaline environment: corn straw (10 g) was subjected to a solution of calcium hydroxide (5 g Ca(OH)_2_ + 130 mL of distilled H_2_O) in a round-bottom flask and then to pressure (0.15 MPa) and thermal (135 °C) treatment in 30 min time [52]. Before thermal hydrolysis, the obtained mass was diluted with water in 1:1.25 ratio. After pretreatment, the slurry was cooled to 80 °C and then activated carbon was added.

The detoxification process was conducted using activated carbon (in 1:5 ratio) in 80 ± 2 °C temperature for 2 h, with continuous rotation at 150 rpm. Then, the lignocellulosic substrate was washed with distilled water (7 L H_2_O/140 mL of the sample) and after filtering, it was dried in 105 °C for 1 h [16].

#### 3.2.3. Enzymatic Hydrolysis

High solids-based enzymatic hydrolysis was carried out at 50 °C for 4 h (pre-saccharification step) and then at 37 °C (SSF process). The enzymatic hydrolysis process was conducted with continuous rotation at 150 rpm, with pH levels in the range of 4.0–6.0. A complex of enzymes was used: cellulase (*Celluclast 1.5 L*, Novozymes Company, Bagsværd, Denmark) and arabanase, β-glucanase, hemicellulase, and xylanase (*Viscozyme Wheat HT,* Novozymes Company, Bagsværd, Denmark). Cellulase is a catalyst for the decomposition of cellulose into glucose, cellobiose, and higher glucose polymers, and its enzyme activity is 700 U/g, 30 U/g s.m.

### 3.3. Bioethanol Production

#### 3.3.1. Microbial Strain

*Saccharomyces cerevisiae* (*S.cerevisiae*) D-2 in the form of yeast cream was used for bioethanol production. The yeast strain D-2 originated from the collection of the Institute of Agricultural and Food Biotechnology. It was stored at 4 °C on the solid YPD culture medium containing (in 1000 mL): yeast extract, 10 g; glucose, 20 g; peptone, 20 g; agar, 20 g (pH 5.0 ± 0.1). D-2 strain is characterized by: alcohol resistance (above 95 g/L EtOH), osmophily (22–24 °Blg), thermophily (38–40 °C), and acidophily (pH 3).

#### 3.3.2. SSF—Simultaneous Saccharification and Fermentation

The studies on obtaining ethanol from lignocellulosic substrate were based on the simultaneous saccharification and fermentation (SSF) method, which is a simultaneous hydrolysis of enzymatic cellulose and fermentation of the obtained sugars [17]. The pre-saccharification time was carried out for 4 h, then the substrate was cooled to 37 °C and inoculated with *S.cerevisiae*. Studies were carried out on a laboratory scale by applying glass flasks (0.5 L) closed with fermentation bungs containing glycerine.

Yeast cream was added to the substrate in the amount of 4 mL/240 mL of the sample and 25 mL/1500 mL of the sample. Each sample contained additionally two flasks of 2.0 L (filled to 1.5 L) destined for distillation after fermentation had been completed (after 72 h) in order to determine by-products.

The alcoholic fermentation was conducted in 38 °C, 40 °C, and 42 °C, under anaerobic stationary conditions for 72 h. Samples for sugars and ethanol analyses were withdrawn periodically. All experiments were performed in triplicate and average values were reported.

### 3.4. Biogas Production

The research was conducted on a laboratory scale in experimental set-up with bioreactor. It was based on anaerobic periodic fermentation, where all the stages of biogas creation happened in the same fermentation container (single phase technology).

Fermentation substrate instilled with methane bacteria was added to the container. During the methane fermentation process, the temperature was kept at a steady level of 37 °C.

The inoculum used in the experiment was an effluent (digestate) from a biogas plant, where manure was used as raw material for biogas production at 36 °C. The inoculum was added at a 10% (*v*/*v*) inoculation ratio to the laboratory bioreactor.

Before the methane fermentation began, the substrate pH was adjusted by using 30% NaOH solution. The pH value was determined during the highest activity of the methane producing bacteria, which was at 7.5 ± 0.7.

The fermentation lasted for 8 days, until a significant decrease in the volume of emitted biogas and its methane content were observed. The cellulose stillage produced during alcoholic fermentation of lignocellulose biomass was treated as a monomaterial in the methane fermentation—as a single source of biogas production.

### 3.5. Analytical Methods

Dry matter (DM) and dry organic matter (DOM) were measured according to standard methods, to measure DM the samples were heated over 2 h at 105 °C, for DOM measurement samples were mashed at 550 °C for 3 h. The content of apparent extract (in °Blg) in fermenting and attenuated mash was determined using the aerometric method. The content of directly reducing sugars in attenuated biomass was measured with the Lane-Eynon method [53].

Effects of the fermentation process were evaluated based on ethanol concentration in the post-fermentation medium. Ethanol concentration (g/L) in fermenting and attenuated mesh was determined using a Carl–Zeiss refractometer and alcohol tables, with an earlier prepared 100 mL sample, using the distillation method. Based on the obtained alcohol concentration, biotechnological indicators of the fermentation were calculated—the ethanol yield from cellulose, actual speed of fermentation, fermentation productivity, and yield [54].

To ensure the control over the course of the methane fermentation, the following indicators were determined during the process [51].

Degree of organic substance decomposition (DOSD) (%):

(1) $$\mathrm{DOSD}\;=\;\frac{S_{D}-S_{O}}{S_{D}}\cdot 100\;(\%)$$

where: S_D_—the dry organic matter of the biomass, before process (kg/m^3^). S_O_—the dry organic matter of the biomass, after processing (kg/m^3^),

Fermentation module (%):

(2) $$M_{f}\;=\;100\;\times \;(1\;-\;\frac{{\mathrm{smo}}_{1}\cdot {\mathrm{smn}}_{0}}{{\mathrm{smo}}_{0}\cdot {\mathrm{smn}}_{1}}\;)$$

where: smo_0_, smo_1_—the dry organic matter of the biomass (%) before and after methane fermentation. smn_0_, smn_1_—the mineral substances of the biomass (%) before and after methane fermentation,

Biogas volume of 1 kg of dry organic matter (L/kg DOM):

G_J_ = G_e._/(Q(S_D_ − S_O_)) (3)

where: G_e_—the amount of the obtained biogas (m^3^). Q—workload of the fermenter (m^3^). S_D_—the dry organic matter of the biomass, before processing (kg/m^3^). S_O_—the dry organic matter of the biomass, after processing (kg/m^3^).

#### Marking Bioethanol and Biogas with Gas Chromatography Method

Marking of chemical contamination in the obtained distillates was performed with capillary gas chromatography method with the use of Hewlett Packard 6890 gas chromatographer with EPC (Electronic Pneumatic Control) unit, flame ionization detector (FID), and Chrompack CP-WAX 57-CB polar capillary column (high polarity polyethylene glycol) with the dimensions of 50 m/320 µm/0.20 µm. A computer analytical station with Hewlett Packard Chem-Station software was used for integrating the signal and for reporting. The chromatographer is equipped with a flame ionization detector that is optimized for capillary columns with EPC unit. A working temperature of 240 °C, nitrogen was used as makeup gas in constant makeup flow mode at 30 mL/min, hydrogen flow at 25 mL/min, air flow at 280 mL/min.

Biogas volume was monitored every day by the water displacement method, and the corresponding cumulative biogas volume was calculated. CH_4_ and CO_2_ were analyzed using a gas chromatograph in isothermal conditions, equipped with thermal conductivity detector (katharometer, Laboratorní přístroje Praha, Czech Republic) with incandescent fiber. Chromosorb P (250–315 µm) was used as a carrier, and it was processed by acid washing. Motor oil (DC 200 type) with 35 × 10^−5^ m^2^/s viscosity was the stationary phase. During the analysis, the temperature of the thermostatic chamber was set to 70 °C and the volume of gas flow was set to 30 mL/min. The detector temperature was set to 150 °C for 60 mA electric current. A stable level of the zero line was achieved with the help of regulation elements for compensation sensitivity. The examination was performed in a 24 h cycle. The amount of emitted gas was constantly monitored.

## 4. Conclusions

As a result of the alcoholic fermentation process of lignocellulosic biomass, bioethanol and cellulose stillage were obtained. After alcoholic fermentation, the remaining cellulose stillage was subjected to methane fermentation.

Based on the SSF process of corn straw, ca. 17 g/L ethanol was obtained, while the ethanol yield amounted to ca. 31 L EtOH/100 kg of cellulose. The cellulose stillage used for the biogas production was characterized by fast digestion, and the efficiency of biogas was ca. 330 L/kg DOM.

Based on the results of the methane fermentation examination, it was determined that the waste made after the production of ethanol can provide biodegradable components for methane bacteria. This results in rapid acid phase and efficient conduction of subsequent stages of methanogenesis, leading to the conception of high-power biogas.

The combination of two technologies, where the waste product of the first is a starting product of the second, creates the possibility for lowering the amount of waste for both regional and national systems. Additionally, the waste product from the biogas plant (post-fermentation) is a fertilizer component. The described combination of technologies for obtaining two biofuels enables the production of wasteless technology.

In addition to the utilization of agricultural waste (straw) during fermentation processes (alcoholic and methane), two types of fuels are produced, i.e., bioethanol and biogas. Those fuels can be used as power in plants which are producing them, and in 80% can be sold to fuel or electrical companies.

## Figures and Tables

**Table 1:** The amount of lignocellulose components in corn straw, before and after chemical pretreatment (135 °C, 0.15 MPa, Ca(OH)_2_) and enzymatic hydrolysis (50 °C, pH 5.0–5.2, 4 h).

Components of Lignoelluloses	Before Pretreatment		After Pretreatment	
	Result (%)	Uncertainty (%)	Result (%)	Uncertainty (%)
Cellulose	11.57	± 0.68	7.76	± 0.43
Hemicellulose	43.21	± 2.41	9.71	± 0.87
Lignin	2.22	± 0.04	1.26	± 0.03
Other (Ash + Extractives)	43.00	± 1.69	81.27	± 3.45

Notes: Table shows mean values and standard deviations. Extractives–non-structural material from biomass (the soluble matter in the residue).

**Table 2:** Parameters describing alcoholic fermentation of corn straw—processing, with detoxification, T = 40 °C, simultaneous saccharification and fermentation (SSF) method.

Parameters	Fermentation Time (h)			
	0	24	48	72
Apparent extract (°Blg)	5.00 ± 0.02	3.80 ± 0.00	2.90 ± 0.00	2.90 ± 0.00
Concentration of ethanol (g/L)	-	14.54 ± 0.38	16.98 ± 0.26	16.98 ± 0.00
Actual extract (°Blg)	5.00 ± 0.02	3.90 ± 0.10	3.20 ± 0.0	3.20 ± 0.00
Reducing sugars in slop (mg/L)	19.10 ± 0.03	6.12 ± 0.00	5.10 ± 0.00	4.90 ± 0.04

Notes: Table shows mean values and standard deviations. The results for 0 h of alcohol concentration were not subjected to the fermentation process. °Blg: degree Balling is the sugar content of 100 g an aqueous solution, comparable scales for indicating sucrose content is the degree Brix (°Bx).

**Table 3:** Biotechnological indicators of corn straw alcoholic fermentation, T = 40 °C, SSF method.

Biotechnological Indicators	Fermentation Time (h)		
	24	48	72
Ethanol yield (L EtOH/100 kg of cellulose)	26.74 ± 1.23	31.25 ± 0.82	31.25 ± 0.21
Speed of fermentation (L EtOH/kg of glucose x h)	10.03 ± 0.46	5.86 ± 0.15	3.91 ± 0.03
Fermentation productivity (L EtOH/L of mash x h)	0.77 ± 0.04	0.45 ± 0.01	0.30 ± 0.00
Fermentation yield (% of theoret.)	37.24 ± 1.72	43.51 ± 1.14	43.51 ± 0.29

Notes: Table shows mean values and standard deviations.

**Table 4:** The amount of by-products obtained in raw spirits during corn straw alcoholic fermentation.

Type of Chemical Compound	Corn Straw	
	Result (g/L EtOH)	Uncertainty (g/L EtOH)
Aldehydes:	0.209	± 0.071
*Acetaldehyde*	0.125	± 0.015
*Propionaldehyde*	0.011	± 0.001
*Furfural*	0.073	± 0.001
Esters	0.018	± 0.003
Methanol	0.001	± 0.000
Higher alcohols:	2.009	± 0.004
*Isoamyl alcohol*	0.417	± 0.065
*n-propanol*	0.436	± 0.013
*Isobutanol*	1.152	± 0.046
*n-butanol*	0.004	± 0.000

**Table 5:** Parameters of periodical (static) fermentation of cellulose stillage, T = 37 °C.

Retention Time (day)	pH	Dry Matter (DM) (g/L)	Dry Organic Matter (g/L DM)	VFA (mg/L)	Daily Biogas Volume (L/L)
0	7.45 ± 0.02	92.0 ± 0.8	66.2 ± 0.6	850.0 ± 33,7	-
1	5.17 ± 0.08	85.9 ± 2.2	54.8 ± 0.1	1904.2 ± 58.1	3.73 ± 0.35
2	6.13 ± 0.12	79.8 ± 2.4	48.1 ± 0.3	960.8 ± 38.9	4.20 ± 0.42
3	6.52 ± 0.15	78.3 ± 1.7	46.5 ± 2.1	578.4 ± 29.9	1.20 ± 0.10
4	6.77 ± 0.09	73.5 ± 0.9	41.5 ± 0.8	520.1 ± 37.8	0.80 ± 0.07
5	6.85 ± 0.05	69.1 ± 1.1	37.0 ± 0.5	548.6 ± 41.8	0.33 ± 0.03
6	6.91 ± 0.06	67.8 ± 0.7	35.7 ± 0.9	556.9 ± 25.8	0.13 ± 0.04
7	6.83 ± 1.10	66.5 ± 2.5	34.2 ± 1.8	523.3 ± 32.7	0.13 ± 0.02
8	7.02 ± 0.08	66.5 ± 1.9	34.2 ± 1.8	580.2 ± 18.8	0.10 ± 0.01

Notes: Table shows mean values and standard deviations.

**Table 6:** Parameters obtained after anaerobic digestion of cellulose stillage. T = 37 °C, pH 7.5, HRT = 8 days.

Parameters	Cellulose Stillage	
	Result	Uncertainty
Degree of organic substance decomposition (DOSD) (%)	48.34	± 2.75
Fermentation module (%)	58.74	± 1.48
Biogas volume from 1 L of cellulose stillage (L/L)	10.63	± 0.49
Biogas volume of 1 kg of dry organic matter (L/kg DOM)	330.0	± 0.0

**Table 7:** Characteristics of the raw material.

Raw Material	Moisture (%)	Dry Matter DM (%)	Dry Organic Matter (% DM)	Mineral Substances (% DM)	Crude Fibre (% DM)
Corn straw	6.8 ± 0.2	93.2 ± 1.1	94.3 ± 1.5	5.7 ± 0.1	39.2 ± 0.7
